# Reanalysis of Urothelial Cancer Chemoimmunotherapy Trials With Differential Censoring

**DOI:** 10.1001/jamanetworkopen.2024.55630

**Published:** 2025-01-22

**Authors:** Tomer Meirson, Jonathan Ofer, Noa Zimhony-Nissim, Avital Bareket-Samish, Gal Markel, Victoria Neiman, Nathan Cherny, Daniel A. Goldstein, Bishal Gyawali, Ian Tannock, Eli Rosenbaum

**Affiliations:** 1 Davidoff Cancer Center, Rabin Medical Center, Petach Tikvah, Israel; 2 Faculty of Medicine, Tel Aviv University, Tel Aviv, Israel; 3 Samueli Integrative Cancer Pioneering Institute, Rabin Medical Center-Beilinson Hospital, Petach Tikva, Israel; 4 BioInsight, Binyamina, Israel; 5 Shaare Zedek Medical Center, Jerusalem, Israel; 6 Queen’s University Cancer Research Institute, Kingston, Ontario, Canada; 7 Division of Medical Oncology, Princess Margaret Cancer Centre, Toronto, Ontario, Canada

## Abstract

**Importance:**

Three similar phase 3 randomized clinical trials have investigated PD-1/PD-L1 (programmed cell death 1 protein/programmed cell death 1 ligand 1) inhibitors in combination with platinum-based chemotherapy vs chemotherapy alone as first-line treatment for advanced urothelial carcinoma (IMvigor130, atezolizumab; KEYNOTE-361, pembrolizumab; and CheckMate901, nivolumab). Only CheckMate901 reported overall survival (OS) benefit for the combination. The reason for these inconsistent results is unclear.

**Objective:**

To explore whether differential censoring—that is, censoring imbalance between the study groups—is a possible explanation for these inconsistent findings.

**Design, Setting, and Participants:**

This comparative effectiveness study involved a censoring analysis of data from IMvigor130, KEYNOTE-361, and CheckMate901, which enrolled patients between 2016 and 2022. Participants included patients in these 3 trials.

**Exposure:**

Participation in 1 of the 3 trials.

**Main Outcomes and Measures:**

The primary outcomes were censoring rates adjusted for treatment effects. Censoring rates were calculated from the Kaplan-Meier (KM) curves. When excess censoring in the control group of open-label trials was found, the hypothesis was that better-performing patients might be dropping out to seek alternative treatments; a sensitivity analysis was conducted in which their survival was assumed to be similar to that of the longest surviving patients in the control group. Treatment effects of the censoring-adjusted KM curves were calculated using the 2-sided log-rank test.

**Results:**

The 3 trials involved a total of 2162 patients (1640 male [76%]; age range, 65-69 years) Analysis of progression-free survival (PFS) curves demonstrated no differential censoring in IMvigor130, but there was more than 30% excess censoring in the chemotherapy-only groups in KEYNOTE-361 and CheckMate901 trials. After sensitivity analysis, the PFS benefit was no longer significant in either study (KEYNOTE-361, adjusted hazard ratio [HR], 1.13 [95% CI, 0.95-1.35]; CheckMate901, adjusted HR, 1.17 [0.96-1.44]). Analysis of OS curves demonstrated no differential censoring in IMvigor130 or KEYNOTE-361, but there was more censoring in the chemotherapy-only group in CheckMate901. After sensitivity analysis, the OS benefit of adding nivolumab to chemotherapy was lost (before adjustment, HR, 0.77 [95% CI, 0.63-0.95]; *P* = .01; adjusted HR, 0.95 [95% CI, 0.77-1.17]; *P* = .64).

**Conclusions and Relevance:**

In this comparative effectiveness study, differential censoring explained the inconsistent results reported in the evaluated trials. The term *perceived-inferiority censoring* is suggested to describe a phenomenon wherein better-performing patients are aware of their treatment and drop out to pursue alternative therapeutic options; it is possible that this occurred in the open-label KEYNOTE-361 and CheckMate901 trials. Such censoring confounds randomization and interpretation of clinical trials, since a larger experimental group is compared with a selected group of controls with poorer prognosis.

## Key Points

**Question:**

Could differential censoring explain the inconsistent results in phase 3 trials investigating the addition of PD-1/PD-L1 (programmed cell death 1 protein/programmed cell death 1 ligand 1) inhibitors to platinum-based chemotherapy as first-line treatment in advanced urothelial carcinoma?

**Findings:**

In this comparative effectiveness study of 2162 patients in the IMvigor130, KEYNOTE-361, and CheckMate901 trials, differential censoring with increased censoring in the chemotherapy-alone group was present for overall survival only in CheckMate-901. After adjusting for it, the significance for the overall survival benefit reported for CheckMate-901 was lost.

**Meaning:**

These findings suggest that differential censoring could explain inconsistent results between 3 similar trials.

## Teaser

This comparative effectiveness study examines whether differential censoring could explain the inconsistent results in trials investigating the addition of programmed cell death 1 protein/programmed cell death 1 ligand 1 inhibitors to platinum-based chemotherapy for advanced urothelial carcinoma.

## Introduction

Three phase 3 randomized clinical trials (RCTs)^1,2,3^ investigated adding PD-1/PD-L1 (programmed cell death 1 protein/programmed cell death 1 ligand 1) inhibitors to platinum-based chemotherapy as first-line treatment in advanced urothelial carcinoma. These studies, which were similar in their design and eligibility criteria, included IMvigor130 (atezolizumab),^1^ KEYNOTE-361 (pembrolizumab),^2^ and CheckMate901 (nivolumab).^3^ All studies reported a statistically significant increase in progression-free survival (PFS) for the combination over chemotherapy alone. Only 1 study (CheckMate901) reported a statistically significant overall survival (OS) benefit, which led to the US Food and Drug Administration’s approval of nivolumab for this indication. The CheckMate901 authors suggested that inconsistency in the OS results might be due to differences in the immunomodulatory effects of cisplatin (the only platinum agent in CheckMate901) vs carboplatin (IMvigor130 and KEYNOTE-361 allowed either agent).^3^ However, exploratory subgroup analyses evaluating the platinum agent used do not support this explanation.^1,2^ The objective of the current study was to explore differential censoring between study groups, as a possible explanation for the inconsistent findings of these RCTs.

## Methods

### Study Design

This is a comparative effectiveness study examining the censoring patterns in the 3 RCTs (IMvigor130, KEYNOTE-361, and CheckMate901).^1,2,3^ In IMvigor130, enrollment occurred between 2016 and 2018, and the median (IQR) follow-up time was 11.8 (6.1-17.2) months; in KEYNOTE-361, enrollment also occurred between 2016 and 2018 (median [IQR] follow-up, 31.7 [27.7-36.0] months). CheckMate901 was the latest trial of the 3 RCTs (enrollment, 2018-2022; median [IQR] follow-up, 33.6 [7.4-62.4] months).^1,2,3^ No institutional review board approval or informed consent was required for this analysis, which used deidentified public domain datasets, in accordance with 45 CFR §46. This study follows International Society for Pharmacoeconomics and Outcomes Research (ISPOR) reporting guidelines.

### Statistical Analysis

The Kaplan-Meier (KM) curves for OS and PFS of the intention-to-treat populations in the 3 trials were extracted and reconstructed using WebPlotDigitizer and reconstructKM in R software version 4.3.2 (R Project for Statistical Computing).^4^ Reverse KM curves were generated by reversing the status of the time-dependent outcome (ie, 1 = censored; 0 = death). Censoring rates were calculated from the reverse KM curves, and hazard ratios (HRs) were calculated using the Cox proportional hazards model. Excess censoring was calculated by subtracting the reverse KM estimate of the intervention group from the control group. Sensitivity analysis was performed according to the observed primary direction of the censoring. When excess censoring in the control group of an open-label trial was found, we hypothesized that the censored patients were better-performing patients and that their distribution of survival was similar to that of the same number of the longest surviving patients who remained in that group. Thus, these patients were iteratively modeled after the longest surviving patients until balance was restored (0% excess censoring). When excess censoring in the intervention group was found, we hypothesized that the censored patients were poorly performing and experienced an event. Alternative sensitivity analyses^5^ entailed balancing the censoring rates between study groups using the same assumptions to restore balance. When excess censoring in the control group was found, we censored the longest-surviving patients in the intervention group, and when excess censoring in the intervention group was found, we censored the patients with the shortest survival time in the control group.

*P *values were calculated using a 2-sided unstratified log-rank test. All analyses were performed in R statistical software version 0.1.0 (R Project for Statistical Computing). *P* < .05 was considered statistically significant.

## Results

The trial design and main patient characteristics of the 3 RCTs are presented in the Table, with additional details presented in eTable 1 in Supplement 1.^1,2,3^ In all studies, the PD-1/PD-L1 inhibitor was added to a regimen containing gemcitabine plus platinum-based chemotherapy. KEYNOTE-361 and CheckMate901 were both open-label studies, whereas investigators and patients participating in IMvigor130 were blinded as to the study drug. In IMvigor130 and KEYNOTE-361, the study included a third group receiving PD-1/PD-L1 inhibitor monotherapy, not captured in the current analysis. Overall, the study design and the patient populations were similar among the 3 RCTs. In all studies, the majority of patients were male (IMvigor130, 636 of 851 patients [75%]; KEYNOTE-361, 534 of 703 patients [76%]; CheckMate901, 470 of 608 patients [77%]), and the median age ranged from 65 to 69 years. Notably, in IMvigor130 and KEYNOTE-361, 6% to 13% of patients had Eastern Cooperative Oncology Group performance status greater than 1, whereas in CheckMate901, such patients constituted less than 1% of the patient population.

**Table.** Summary of the 3 Evaluated Clinical Trials

Characteristic	IMvigor130^1^		KEYNOTE-361^2^		CheckMate901^3^	
	Atezolizumab plus PBC	PBC	Pembrolizumab plus PBC	PBC	Nivolumab plus gemcitabine-cisplatin	Gemcitabine-cisplatin alone
Design	Blinded for atezolizumab		Open label		Open label	
Follow-up, median (IQR), mo	11.8 (6.1-17.2)		31.7 (27.7-36.0)		33.6 (7.4-62.4)	
Patients, No.	451	400	351	352	304	304
Treatment	Atezolizumab plus gemcitabine with carboplatin or cisplatin	Placebo plus gemcitabine with carboplatin or cisplatin	Pembrolizumab plus gemcitabine with carboplatin or cisplatin	Gemcitabine carboplatin or cisplatin	Nivolumab plus gemcitabine with cisplatin	Gemcitabine with cisplatin
Patient sex, No. (%)						
Male	338 (75)	298 (75)	272 (78)	262 (74)	236 (78)	234 (77)
Female	113 (25)	102 (25)	79 (22)	90 (26)	68 (22)	70 (23)
Age, median (IQR), y	69 (62-75)	67 (61-73)	69 (62-75)	69 (61-75)	65 (32-86)^a^	65 (35-85)^a^
Primary tumor site, No. (%)						
Bladder	312 (69)	293 (73)	Lower tract UC: 287 (82)	Lower tract UC: 270 (77)	235 (77)	219 (72)
Urethra	10 (2)	5 (1)			0	0
Renal pelvis	64 (14)	58 (15)	Upper tract UC: 64 (18)	Upper tract UC: 82 (23)	33 (11)	44 (14)
Ureter	59 (13)	42 (11)			0	0
Other or unknown	6 (1)	2 (1)	0	0	36 (12)	41 (13)
Reported/reconstructed, HR (95% CI)						
Progression-free survival	0.82 (0.70-0.96)/0.79 (0.67-0.92)		0.78 (0.65-0.93)/0.77 (0.65-0.92)		0.72 (0.59-0.88)/0.69 (0.56-0.85)	
Overall survival	0.83 (0.69-1.0)/0.84 (0.69-1.0)		0.86 (0.72-1.0)/0.86 (0.72-1.0)		0.78 (0.63-0.96)/0.77 (0.63-0.95)	

Abbreviations: HR, hazard ratio; PBC, platinum-based chemotherapy; UC, urothelial carcinoma.

^a^ These data are ranges (minimum-maximum) not IQRs.

**Figure 1.**
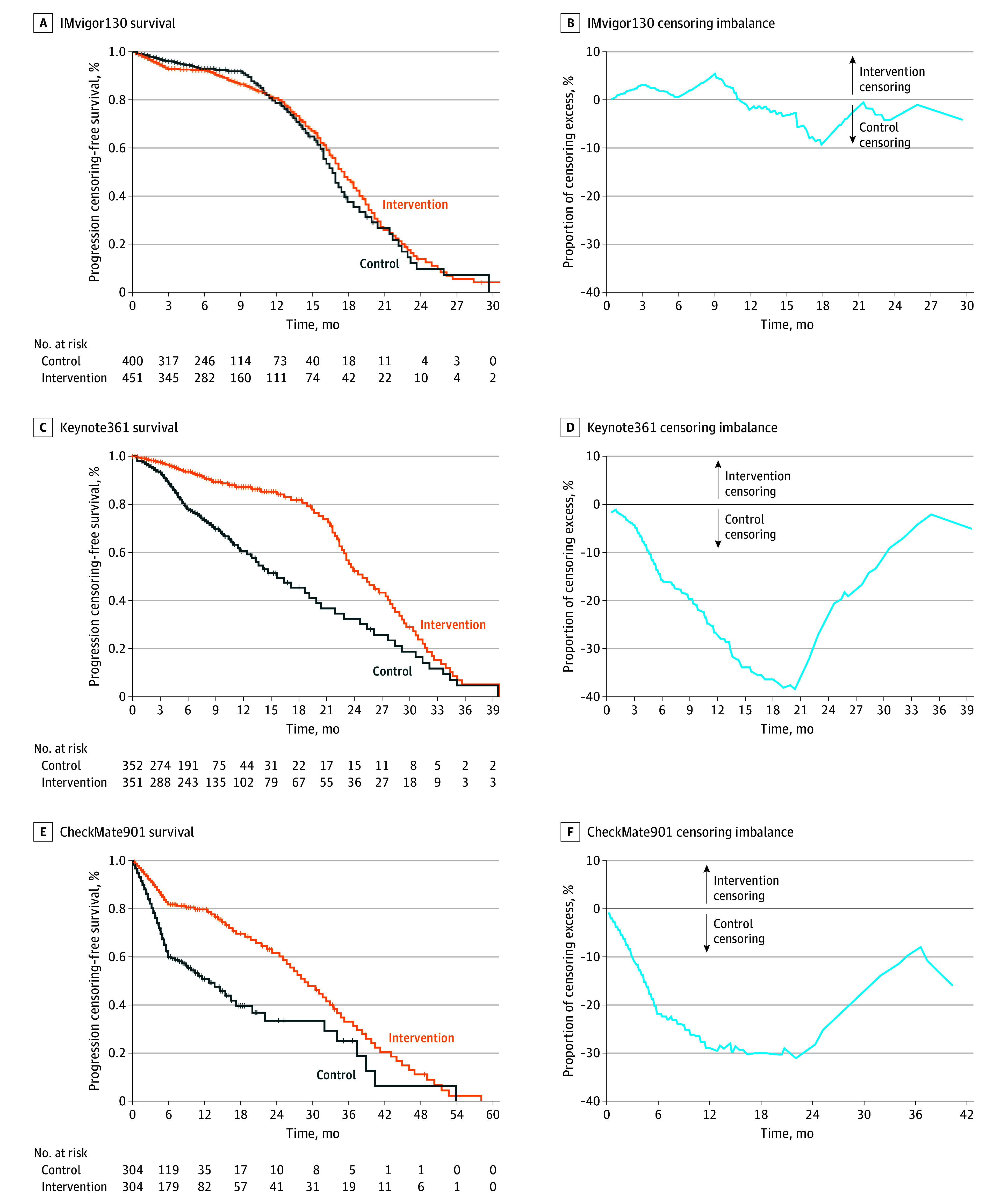
Reverse Kaplan-Meier Curves and Censoring Imbalance Analysis for the Progression-Free Survival End Point Graphs show data for IMvigor130 (A and B), KEYNOTE-361 (C and D), and CheckMate901 (E and F).

**Figure 2.**
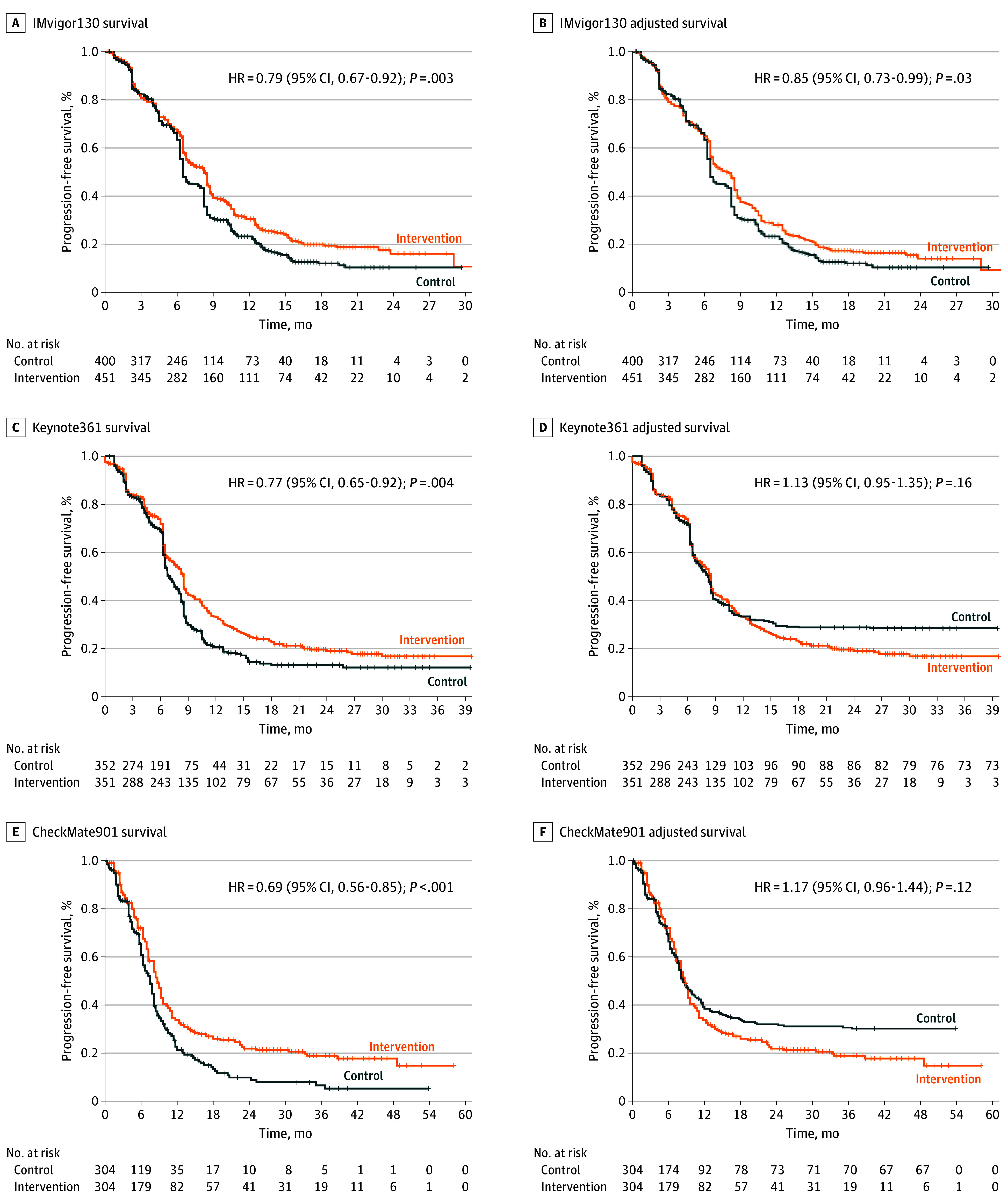
Reconstructed and Adjusted Kaplan-Meier Curves for the Progression-Free Survival End Point Graphs show data for IMvigor130 (A and B), KEYNOTE-361 (C and D), and CheckMate901 (E and F). The sensitivity analysis was adjusted for excess censoring in the intervention group for IMvigor130, and for excess censoring in the control group for KEYNOTE-361 and CheckMate901. HR indicates hazard ratio.

The reconstruction of the KM plots from the published curves was robust, because the reconstructed HR values were very similar to those presented in the reports (Table). For each study, the censoring in each group for PFS and OS at the time point when the maximal excess censoring in the control group and the intervention group is presented in eTable 2 in Supplement 1. For the double-blinded IMvigor130 study, for PFS, at the time point of maximal excess censoring in the control group, a mean (SE) of 62.4% (16.7%) of patients in the chemotherapy-only group and a mean (SE) of 53.1% (10.1%) of patients in the chemotherapy plus atezolizumab group were censored, resulting in excess censoring in the control group of 9.3% (eTable 2 in Supplement 1 and Figures 1A and 1B). After adjustment of the KM curves, the PFS benefit of adding atezolizumab to platinum-based chemotherapy remained statistically significant (HR, 0.79 [95% CI, 0.67-0.92]; *P* = .003; adjusted HR, 0.85 [95% CI, 0.73-0.99]; *P* = .03) (Figures 2A and 2B).

In contrast, more than 30% censoring excess in the control group was observed in the PFS curves for both open-label studies, KEYNOTE-361 and CheckMate901. In KEYNOTE-361, at the time point of maximal excess censoring of the control group, a mean (SE) of 63.6% (16.0%) of patients in the chemotherapy-only group and a mean (SE) of 24.9% (5.5%) of patients in the chemotherapy plus pembrolizumab group were censored, resulting in excess censoring in the control group of 38.4% (eTable 2 in Supplement 1 and Figures 1C and 1D). In CheckMate901, at the time of the maximal excess censoring, the censoring values were a mean (SE) of 66.6% (17.6%) for the chemotherapy-only group and a mean (SE) of 35.5% (6.8%) for the chemotherapy plus nivolumab group, resulting in excess censoring in the control group of 31.1% (eTable 2 in Supplement 1 and Figures 1E and 1F). After sensitivity analysis, the PFS benefit was no longer significant for either pembrolizumab (KEYNOTE-361, HR, 0.77 [95% CI, 0.65-0.92]; *P* = .004; adjusted HR, 1.13 [95% CI, 0.95-1.35]; *P* = .16) or nivolumab (CheckMate901, HR, 0.69 [95% CI, 0.56-0.85]; *P* < .001; adjusted HR, 1.17 [95% CI, 0.96-1.44]; *P* = .12) (Figures 2C-2F).

**Figure 3.**
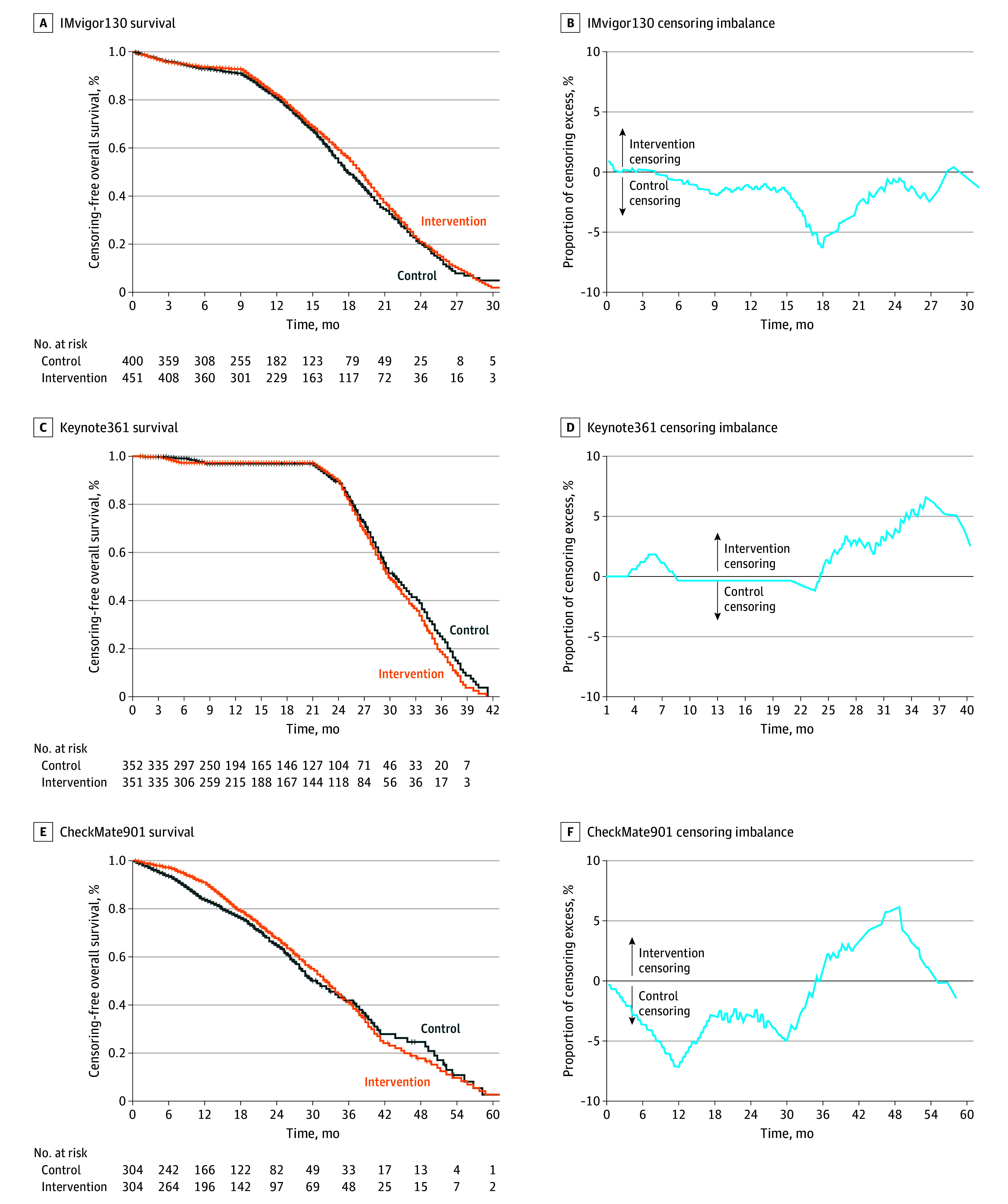
Reverse Kaplan-Meier Curves and Censoring Imbalance Analysis for the Overall Survival End Point Graphs show data for IMvigor130 (A and B), KEYNOTE-361 (C and D), and CheckMate901 (E and F).

**Figure 4.**
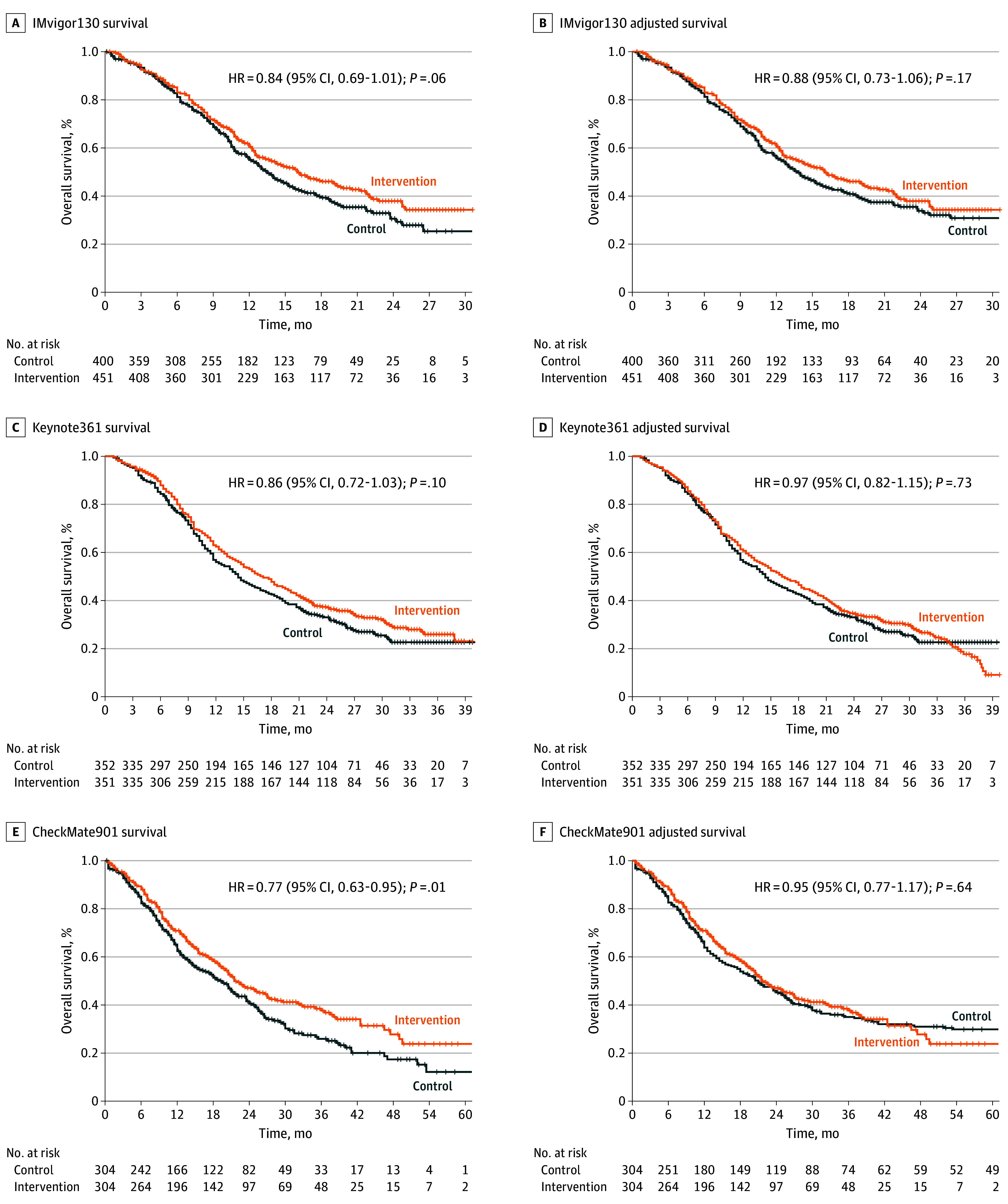
Reconstructed and Adjusted Kaplan-Meier Curves for the Overall Survival End Point Graphs show data for IMvigor130 (A and B), KEYNOTE-361 (C and D), and CheckMate901 (E and F). The sensitivity analysis was adjusted for excess censoring in the control group for IMvigor130 and CheckMate901, and for excess censoring in the intervention group for KEYNOTE-361. HR indicates hazard ratio.

The KM curves for OS in IMvigor130 and KEYNOTE-361 showed only small imbalances in censoring between the study groups for most of the follow-up duration, but there were imbalances in censoring near the end of the follow-up (eTable 2 in Supplement 1 and Figures 3A-3D). In both studies, the HRs demonstrated no OS benefit for adding the PD-1/PD-L1 inhibitor to platinum-based chemotherapy, and this observation remained after adjustment for the differential censoring (Figures 4A-4D).

In contrast, in CheckMate901, differential censoring was observed in both directions (maximal excess censoring in the control group at 12 months, 7.1%; in the intervention group at 49 months, 6.1%) (eTable 2 in Supplement 1 and Figures 3E and 3F). Consequently, after sensitivity analysis, the OS benefit of adding nivolumab to chemotherapy was no longer statistically significant (HR, 0.77 [95% CI, 0.63-0.95]; *P* = .01; adjusted HR, 0.95 [95% CI, 0.77-1.17]; *P* = .64) (Figures 4E and 4F). In CheckMate901, where each group included 304 patients, 172 deaths (56.6%) were reported in the intervention group vs 193 (63.5%) in the chemotherapy only group, reflecting a difference of 6.9% in favor of the intervention group. Sensitivity analyses for PFS and OS were also performed using an alternative approach with similar results (eFigure 1 and eFigure 2 in Supplement 1).

## Discussion

Until recently, platinum-based chemotherapy was the standard-of-care as first-line therapy in advanced urothelial carcinoma. The trials analyzed in the current comparative effectiveness study investigated adding immunotherapy to platinum-based chemotherapy in this setting. Although enfortumab vedotin in combination with PD-1 inhibitor has become the standard treatment,^6,7^ the combination of nivolumab with chemotherapy constitutes the treatment of choice in many countries because the enfortumab vedotin–immunotherapy combination is not accessible owing to its high cost. Furthermore, even when this combination is accessible, there are some patients not suitable for enfortumab because of its specific toxic effects (eg, severe diabetes).^8^

This study used recent methods and approaches developed for analyzing differential censoring^5,9,10,11,12,13,14,15^ and identified imbalanced censoring for both PFS and OS in CheckMate901, with more censoring in the chemotherapy alone group. Our sensitivity analysis indicates that differential censoring could explain away the observed PFS and OS benefit of adding immunotherapy to platinum-based chemotherapy. Although the difference in OS excess censoring seems small (7.1%), it is important relative to the difference in the number of deaths (6.9%). Differential censoring was also identified for PFS in KEYNOTE-361, which could have led to the loss of the reported statistically significant difference in PFS of the combination over chemotherapy alone.

A possible reason for excess censoring in the control group of an unblinded study is that patients randomized to the control group may perceive their treatment as inferior and may have a lower threshold to drop out of the study. It is reasonable to hypothesize that patients who drop out for this reason are likely to be those who are more informed; they may have better performance status and prognosis. This could confound random allocation, since a larger proportion of those randomized to experimental therapy is compared with a smaller group of controls with poorer prognosis, thereby biasing the results of the trial in favor of the experimental therapy. We suggest the term *perceived-inferiority censoring* to describe the phenomenon in which patients are aware of their treatment and drop out to pursue alternative therapeutic options.

The open-label design for both KEYNOTE-361 and CheckMate901 and the conduct of these studies after the initial presentation of the IMvigor130 data, which demonstrated signals for efficacy (greater PFS), suggest that the differential censoring evident in the PFS curves of these trials might be due to perceived-inferiority censoring. Furthermore, CheckMate901 had imbalanced censoring for OS, where unlike PFS, no imaging and patient cooperation is required to document an event. This could be linked to less-rigorous follow-up of patients who dropped out. A sensitivity analysis to determine the possible influence of censoring should be mandated when reporting the results of clinical trials.

The impact of censoring imbalance on clinical trial interpretation is becoming increasingly recognized and affects regulatory and reimbursement decisions. For example, in the 2019 Food and Drug Administration review of quizartinib for the treatment of adult patients with relapsed or refractory *FLT3*-internal tandem duplication–positive acute myeloid leukemia, early excess censoring (>10%) observed in the placebo group was noted and the drug was not approved then, in part because of concerns that the imbalanced censoring contributed to the OS advantage.^16^

### Limitations

Our study is limited by its dependence on reconstructed data derived from the published KM curves. Therefore, the analysis was univariable and unstratified. Nonetheless, the original and the reconstructed HRs were very similar. The nature of the censoring was not available; thus, the current study can provide only plausible hypotheses. The uncertainty introduced by multiple imputation was not addressed in the current analysis, and this is a limitation of our study. However, for the objective of the current analysis, which was to demonstrate how the results may change compared with the original analysis, the point estimates suffice as they provide most of the information.

## Conclusions

In conclusion, differential censoring may explain the inconsistent results reported in the 3 phase 3 trials evaluating immunotherapy with platinum chemotherapy for urothelial cancer. Perceived-inferiority censoring confounds randomization and interpretation of clinical trial data.

## Notes

Supplement 1. **eTable 1.** Patient characteristics in the 3 evaluated clinical trials **eTable 2.** Censoring values by study and endpoint at the timepoints with maximal differential censoring. **eFigure 1.** Adjusted Kaplan-Meier plots for the progression-free survival endpoint for IMvigor130 (A), KEYNOTE-361 (B), and CheckMate901 (C) using the alternative sensitivity analysis approach **eFigure 2.** Adjusted Kaplan-Meier plots for the overall survival endpoint for IMvigor130 (A), KEYNOTE-361 (B), and CheckMate901 (C) using the alternative sensitivity analysis approach
eReferences

Supplement 2.
Data Sharing Statement
