# Supplementary material for: Reanalysis of Urothelial Cancer Chemoimmunotherapy Trials With Differential Censoring
Source: JAMA Netw Open. 2025 Jan 22;8(1):e2455630. doi: 10.1001/jamanetworkopen.2024.55630 (PMC11755191; doi:10.1001/jamanetworkopen.2024.55630)
Supplement: Supplement 1. — eTable 1. Patient characteristics in the 3 evaluated clinical trials eTable 2. Censoring values by study and endpoint at the timepoints with maximal differential censoring. eFigure 1. Adjusted Kaplan-Meier plots for the progression-free survival endpoint for IMvigor130 (A), KEYNOTE-361 (B), and CheckMate901 (C) using the alternative sensitivity analysis approach eFigure 2. Adjusted Kaplan-Meier plots for the overall survival endpoint for IMvigor130 (A), KEYNOTE-361 (B), and CheckMate901 (C) using the alternative sensitivity analysis approach eReferences [file jamanetwopen-e2455630-s001.pdf]

Supplemental Online Content

Meirson T, Ofer J, Zimhony N, et al. Reanalysis of urothelial cancer chemoimmunotherapy trials with differential censoring. *JAMA Netw Open*. 2025;8(1):e2455630. doi:10.1001/jamanetworkopen.2024.55630

eTable 1. Patient Characteristics in the 3 Evaluated Clinical Trials
eTable 2. Censoring values by study and endpoint at the timepoints with maximal differential censoring.
eFigure 1. Adjusted Kaplan-Meier plots for the progression-free survival endpoint for IMvigor130 (A), KEYNOTE-361 (B), and CheckMate901 (C) using the alternative sensitivity analysis approach
eFigure 2. Adjusted Kaplan-Meier plots for the overall survival endpoint for IMvigor130 (A), KEYNOTE-361 (B), and CheckMate901 (C) using the alternative sensitivity analysis approach
eReferences

This supplemental material has been provided by the authors to give readers additional information about their work.

© 2025 Meirson T et al. *JAMA Network Open*.

eTable 1. Patient Characteristics in the 3 Evaluated Clinical Trials.

	IMVigor130[1]		KEYNOTE-361[2]		CheckMate901[3]	
	**Atezolizumab+ platinum-based chemotherapy n = 451**	**Platinum-based chemotherapy n = 400**	**Pembrolizumab+ platinum-based chemotherapy n = 351**	**Platinum-based chemotherapy n = 352**	**Nivolumab + gemcitabine-cisplatin n = 304**	**Gemcitabine-cisplatin alone n = 304**
Geographic distribution	35 countries, 6 continents (Africa, Australia, Asia, Europe, North America, and South America)[a]		21 countries, 5 continents (Africa, Asia, Europe, North America, and South America)[a]		Europe (45.4%), Asia (21.9%), USA (6.6%), other (26.2%)	
Race, n (%)						
White	346 (77)	304 (76)	NA	NA	211 (69)	225 (74)
Asian	90 (20)	85 (21)	NA	NA	75 (25)	63 (21)
American Indian or Alaska Native	4 (1)	3 (1)	NA	NA	1 (0.3)	1 (0.3)
Black/African American	6 (1)	1 (<1)	NA	NA	0	2 (0.7)
Other/unknown	5 (1)	7 (2)	NA	NA	17 (6)	13 (4)
ECOG performance status score, n (%)						
0	182 (40)	173 (43)	150 (43)	168 (48)	162 (53)	162 (53)
1	209 (46)	187 (47)	178 (51)	162 (46)	140 (46)	142 (47)
>1	60 (13)	40 (10)	23 (7)	22 (6)	2 (0.7)	0
Tumor PD-L1 expression, n (%)						
≥1%	303 (67)	270 (68)	CPS≥10, 159 (45)	CPS≥10, 158 (45)	111 (37)	110 (36)
<1%	148 (33)	130 (33)	CPS<10, 192 (55)	CPS<10, 194 (55)	193 (63)	194 (64)
Platinum agent received, n (%)	Carboplatin: 314 (70) Cisplatin: 137 (30)	Carboplatin: 264 (66) Cisplatin: 136 (34)	Carboplatin: 195 (56) Cisplatin: 156 (44)	Carboplatin: 196 (56) Cisplatin: 156 (44)	Cisplatin: 304 (100)	Cisplatin: 304 (100)

[a] Geographic distribution was not provided.

Abbreviations: CPS, combined positive score; ECOG, Eastern Cooperative Oncology Group; PD-L1, programmed death ligand.

© 2025 Meirson T et al. *JAMA Network Open.*

eTable 2. Censoring values by study and endpoint at the timepoints with maximal differential censoring.

	Excess censoring in the control arm				Excess censoring in the intervention arm			
	Timepoint, month	control arm censoring (SE), %	Intervention arm censoring (SE), %	Absolute difference in censoring, %	Timepoint, month	Intervention arm censoring (SE), %	Control arm censoring (SE), %	Absolute difference in censoring, %
	**Progression-free survival**							
**IMvigor130**[1]	18	62.4 (16.7)	53.1 (10.1)	9.3	9	13.6 (2.3)	8.2 (1.7)	5.4
**KEYNOTE-361**[2]	20	63.3 (16.0)	24.9 (5.5)	38.4	1	–	–	–
**CheckMate901**[3]	22	66.6 (17.6)	35.5 (6.8)	31.1	0.15	–	–	–
	**Overall survival**							
**IMvigor130**[1]	18	50.4 (7.3)	44.1 (5.6)	6.3	0.13	1.1 (0.4)	0.2 (0.2)	0.9
**KEYNOTE-361**[2]	24	10.5 (2.8)	9.3 (2.5)	1.2	36	80.2 (20.1)	73.6 (18.0)	6.6
**CheckMate901**[3]	12	16.2 (2.8)	9.0 (2.0)	7.1	49	83.4 (21.7)	77.3 (20.7)	6.1

CT, chemotherapy; OS, overall survival; PFS, progression-free survival; SE, standard error.

© 2025 Meirson T et al. *JAMA Network Open.*

eFigure 1. Adjusted Kaplan-Meier plots for the progression-free survival endpoint for IMvigor130 (A), KEYNOTE-361 (B), and CheckMate901 (C) using the alternative sensitivity analysis approach. The hazard ratio (HR), 95% CI, and the p-value are included in each graph. The numbers below each panel represent the number of patients at risk.

A IMvigor130

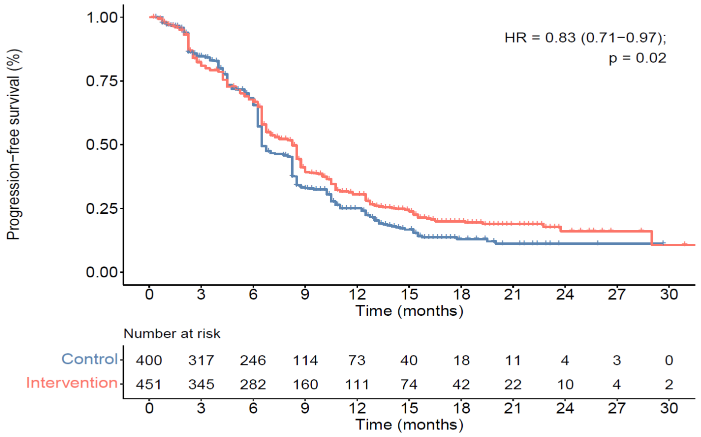

B Keynote361

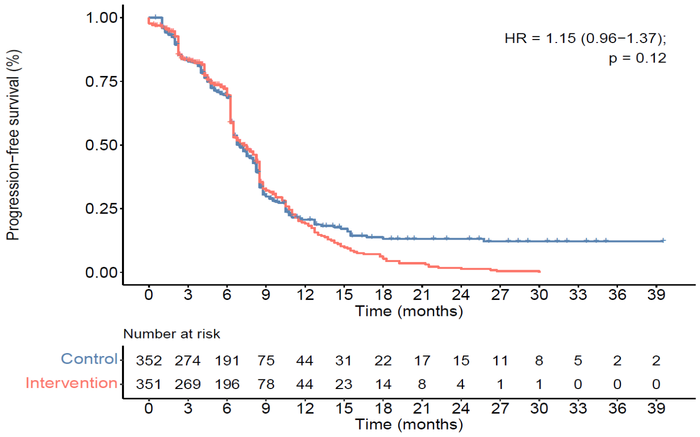

C CheckMate901

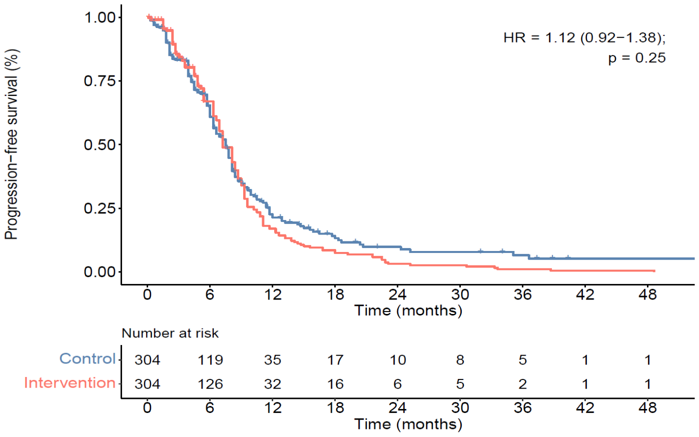

eFigure 2. Adjusted Kaplan-Meier plots for the overall survival endpoint for IMvigor130 (A), KEYNOTE-361 (B), and CheckMate901 (C) using the alternative sensitivity analysis approach. The hazard ratio (HR), 95% CI, and the p-value are included in each graph. The numbers below each panel represent the number of patients at risk.

A IMvigor130

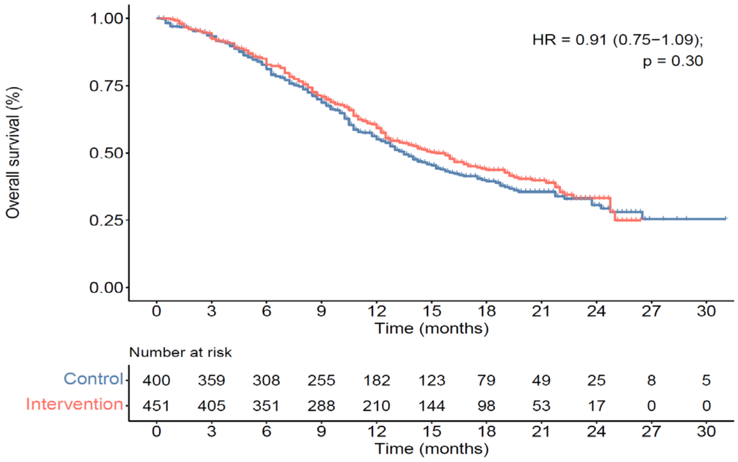

B Keynote361

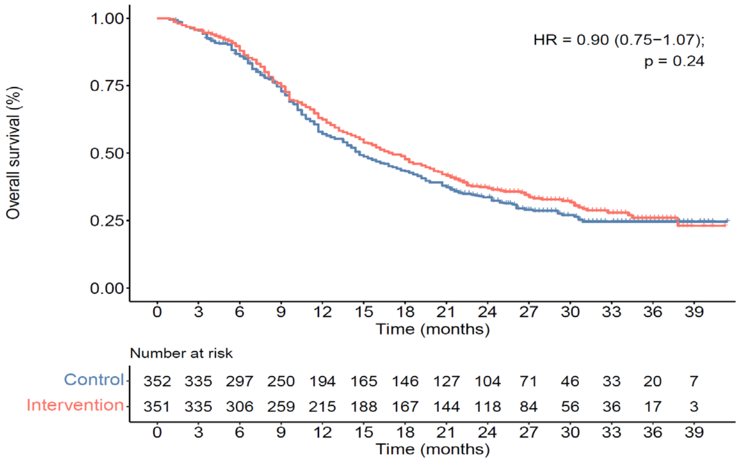

C CheckMate901

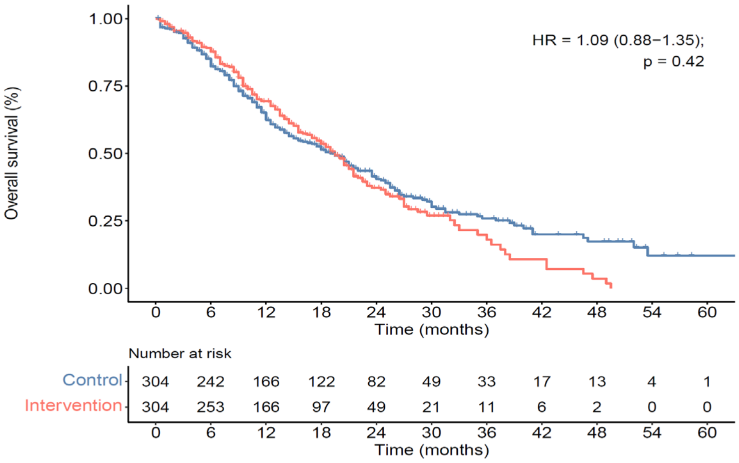

© 2025 Meirson T et al. *JAMA Network Open.*

eReferences

1. Galsky MD, Arija JAA, Bamias A, et al. Atezolizumab with or without chemotherapy in metastatic urothelial cancer (IMvigor130): a multicentre, randomised, placebo-controlled phase 3 trial. *Lancet*. 2020;395(10236):1547-1557. doi:10.1016/S0140-6736(20)30230-0

2. Powles T, Csoszi T, Ozguroglu M, et al. Pembrolizumab alone or combined with chemotherapy versus chemotherapy as first-line therapy for advanced urothelial carcinoma (KEYNOTE-361): a randomised, open-label, phase 3 trial. *Lancet Oncol*. 2021;22(7):931-945. doi:10.1016/S1470-2045(21)00152-2

3. van der Heijden MS, Sonpavde G, Powles T, et al. Nivolumab plus gemcitabine-cisplatin in advanced urothelial carcinoma. *N Engl J Med*. 2023;389(19):1778-1789. doi:10.1056/NEJMoa2309863

© 2025 Meirson T et al. *JAMA Network Open.*
