# Supplementary material for: Reanalysis of Urothelial Cancer Chemoimmunotherapy Trials With Differential Censoring
Source: JAMA Netw Open. 2025 Jan 22;8(1):e2455630. doi: 10.1001/jamanetworkopen.2024.55630 (PMC11755191; doi:10.1001/jamanetworkopen.2024.55630)
Supplement: Supplement 2. — Data Sharing Statement [file jamanetwopen-e2455630-s002.pdf]

Data Sharing Statement

Meirson. Reanalysis of Urothelial Cancer Chemoimmunotherapy Trials With Differential Censoring. *JAMA Netw Open*. Published January 22, 2025.
doi:10.1001/jamanetworkopen.2024.55630

Data
Data available: Yes
Data types: Other (please specify)
Additional Information: The data that support the findings of this study are available from the corresponding author upon request. Email: Tomermrsn@gmail.com
How to access data: The data that support the findings of this study are available from the corresponding author upon request. Email: Tomermrsn@gmail.com
When available: With publication

Supporting Documents
Document types: Statistical/analytic code
How to access documents: The data that support the findings of this study are available from the corresponding author upon request. Tomermrsn@gmail.com
When available: With publication

Additional Information
Who can access the data: Anyone requesting the data
Types of analyses: Statistical/analytic code
Mechanisms of data availability: No specific restrictions
